# Impact of Discontinuation of Contact Precautions on Healthcare-associated Extended Spectrum β-Lactamase-Producing *Escherichia coli* in a Large Tertiary Care Hospital: An Interrupted Time-series Analysis

**DOI:** 10.1093/cid/ciaf594

**Published:** 2025-10-29

**Authors:** Stefano Musumeci, Rebecca Grant, Jonathan Sobel, Abdessalam Cherkaoui, Niccolò Buetti, Stephan Harbarth

**Affiliations:** Division of Infectious Diseases, Geneva University Hospitals and Faculty of Medicine, Geneva, Switzerland; Infection Control Program and WHO Collaborating Center, Geneva University Hospitals and Faculty of Medicine, Geneva, Switzerland; Infection Control Program and WHO Collaborating Center, Geneva University Hospitals and Faculty of Medicine, Geneva, Switzerland; Infection Control Program and WHO Collaborating Center, Geneva University Hospitals and Faculty of Medicine, Geneva, Switzerland; Bacteriology Laboratory, Geneva University Hospitals and Faculty of Medicine, Geneva, Switzerland; Infection Control Program and WHO Collaborating Center, Geneva University Hospitals and Faculty of Medicine, Geneva, Switzerland; Infection Control Program and WHO Collaborating Center, Geneva University Hospitals and Faculty of Medicine, Geneva, Switzerland

**Keywords:** ESBL-producing *Escherichia coli*, contact precautions, healthcare-associated infections, multidrug resistant organisms

## Abstract

At Geneva University Hospitals, contact precautions for ESBL-producing *Escherichia coli* were discontinued in 2019. Among 2336 hospital-associated cases from 2016 to 2024, incidence was 3.1 per 10 000 patient days at baseline and showed no significant increase after discontinuation (slope change −0.04, *P* = .14). Results were consistent when limited to clinical isolates.

Enterobacterales can acquire genes encoding various extended-spectrum β-lactamases (ESBLs), which confer resistance to a wide range of β-lactam antibiotics, limiting treatment options. Initially, contact precautions were recommended for patients colonized or infected with ESBL-producing Enterobacterales to prevent further transmission within healthcare settings [1, 2]. In addition to standard infection control measures, contact precautions require patients to be in single rooms or with adequate spatial separation between beds in shared rooms, the wearing of gloves and gowns by healthcare workers for all interactions with the patient or the patient's direct environment, and enhanced environmental cleaning and disinfection.

However, accumulating evidence indicates that contact precautions may be safely discontinued for patients colonized or infected with ESBL-producing *Escherichia coli* without increasing healthcare-associated transmission. A cluster-randomized crossover trial found that contact precautions offered no additional benefit over standard precautions in limiting the spread of ESBL-Enterobacterales in noncritical care wards [3]. Similarly, an observational study comparing two hospitals with different infection control policies suggested that contact isolation measures were ineffective in limiting the spread of ESBL-producing *E. coli* [4]. In a quasi-experimental study, contact precautions were found to have no impact on nosocomial acquisition of ESBL organisms [5]. The principal arguments in support of and against the use of contact precautions for ESBL-producing *E. coli* have previously been highlighted in this journal [6].

In Switzerland, as community acquisition often outweighs healthcare-related acquisition of ESBL-producing *E. coli* and as population-based incidence rates are unlikely to be influenced by contact precautions in hospitals, healthcare facilities decided to discontinue contact precautions for patients colonized or infected with ESBL-producing *E. coli* [7]. At Geneva University Hospitals, the largest tertiary care center in Switzerland, contact precautions for ESBL-producing *E. coli* were discontinued as of 1 January 2019.

We therefore conducted a retrospective time-series analysis of inpatients at Geneva University Hospitals (HUG) from 1 September 2016 to 31 December 2024 to determine the impact of the discontinuation of contact precautions on the incidence of healthcare-associated ESBL-producing *E. coli*.

## METHODS

### Study Design

As part of its institutional mandate, the Infection Control Program at HUG conducts routine epidemiological surveillance of antibiotic-resistant bacteria among hospitalized patients, including ESBL-producing *E. coli.* During the period 1 September 2016 to 31 December 2024, the indications for screening for antibiotic-resistant bacteria were for the following inpatients: (1) with previously documented antibiotic-resistant bacteria colonization or infection; (2) who were transferred directly from another hospital, (3) who had been in contact with a patient who had been identified with antibiotic-resistant bacteria, or (4) who were in certain high-risk wards (adult intensive care unit, hemato-oncology, septic orthopedics) [8]. For this analysis, no additional patient sampling or data collection were performed beyond routine clinical and infection prevention procedures.

We included all inpatients without previously documented ESBL-producing *E. coli* colonization (rectal swabs, axillary swabs or fecal samples) or infection (any clinical specimen except rectal swabs, axillary swabs or fecal samples) and for whom ESBL-producing *E. coli* was detected for the first time on a sample collected more than 48 hours from the patient's admission during the study period. For patients with multiple positive samples, only the earliest detection was retained, thereby avoiding recurrent detections of previously colonized or infected individuals.

### Laboratory Analyses

Bacterial identification was carried out by matrix-assisted laser desorption ionization–time of flight mass spectrometry (MALDI-TOF/MS) (MBT Compass 4.1, library version 11.0 [11′410 spectra], Bruker Daltonics, Bremen, Germany) according to the manufacturer's instructions. The antimicrobial susceptibility testing and the ESBL screening were performed by a fully automated disk diffusion method [9] according to EUCAST recommendations. ESBL was confirmed phenotypically by double-disc synergy tests (DDST20) using Mueller Hinton E agar (MHE) (bioMérieux). Briefly, the amoxicillin–clavulanate disk (AMC) was automatically placed at 20 mm, center to center, of ceftriaxone (CRO) disk, and cefepime (FEP) disk. The isolate was considered to be an ESBL producer when the inhibition zone around the CRO and/or FEP disks was enhanced. In doubtful cases, the ESBL & AmpC Screen Kit 98 008 (Rosco Diagnostica) was used as a second line confirmatory test [10].

### Statistical Analyses

We calculated the monthly incidence of healthcare-associated ESBL-producing *E. coli* colonization or infection, expressed as the number of new cases per month per 10 000 patient days. We considered monthly incidence prior to and after the discontinuation of contact precautions. In addition, we considered four distinct periods: 1 September 2016 to 31 December 2018 during which contact precautions were recommended for patients colonized or infected with ESBL-producing *E. coli* (*Period 1*, baseline); 1 January 2019 to 29 February 2020 during which contact precautions were no longer recommended for patients colonized with ESBL-producing *E. coli* (*Period 2*, discontinued contact precautions); 1 March 2020 to 31 December 2021 during which the hospital system was often under high strain (*Period 3*, early COVID-19 pandemic); 1 January 2022 to 31 December 2024 during which Omicron was the predominant SARS-CoV-2 variant (*Period 4*, later COVID-19 pandemic).

We conducted an interrupted time-series regression to assess changes in incidence rates of healthcare-associated ESBL-producing *E. coli*. Linear models were fitted with monthly incidence per 10 000 patient days as the outcome, with period (categorical) and time (continuous) as predictors. Model assumptions were checked by visual inspection of residuals, autocorrelation and the Breusch–Pagan test for heteroskedasticity. No evidence of autocorrelation or heteroskedasticity was detected, so no correlation or variance structure was applied. In sensitivity analyses, we restricted our analyses to (1) clinical samples, defined as the detection of ESBL-producing *E. coli* more than 48 hours from the patient's admission on any clinical specimen type except rectal swabs, axillary swabs or fecal samples, and (2) the detection of ESBL-producing *E. coli* in wards in which systematic, weekly screening of all hospitalized patients was conducted throughout the study period: adult intensive care, hemato-oncology and septic orthopedics.

All analyses were performed in R version 4.3.3 (R Foundation, Vienna, Austria) using the tidyverse, tseries, and lmtest packages.

## RESULTS

Between 1 September 2016 and 31 December 2024, there were 2336 incident cases of healthcare-associated ESBL-producing *E. coli* detected among inpatients: 667 detected during Period 1; 337 during Period 2; 448 during Period 3 and 884 during Period 4. Supplementary Figure 1 shows the incidence of healthcare-associated ESBL-producing *E. coli* per 10 000 patient days during each period.

**Figure 1.**
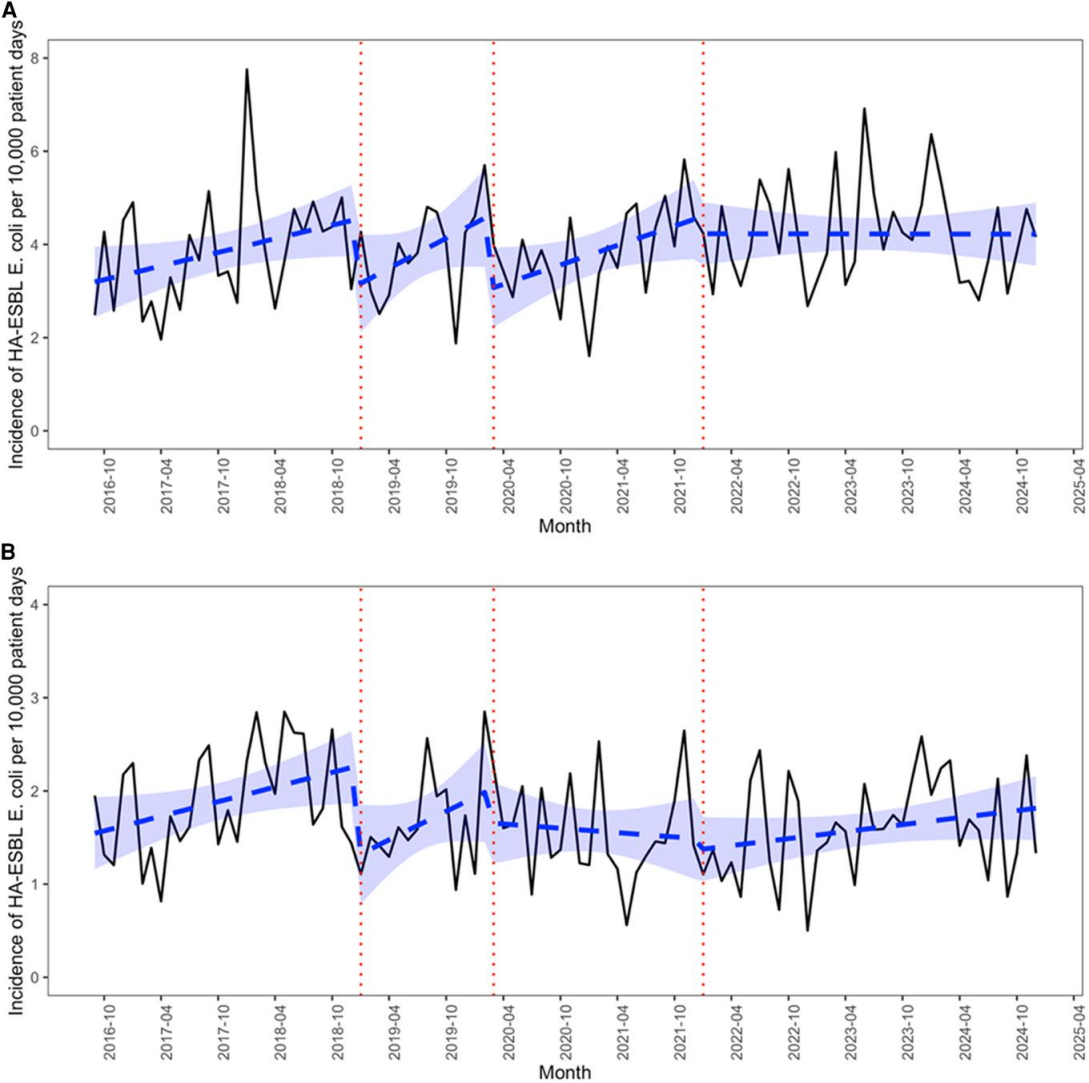
Observed and fitted incidence of healthcare-associated ESBL-producing *E. coli* by period per 10 000 patient days on all samples (*A*) and only on clinical samples (*B*). The red vertical lines distinguish each period: Period 1 (baseline), Period 2 (discontinued contact precautions), Period 3 (early COVID-19 pandemic) and Period 4 (later COVID-19 pandemic). The shaded area indicates the 95%CI of the fitted incidence. Abbreviations: CI, confidence interval; COVID-19, coronavirus 2019; *E. coli*, *Escherichia coli*; ESBLs, extended-spectrum β-lactamases.

At the start of Period 1, the incidence of healthcare-associated ESBL-producing *E. coli* was 3.1 (95%CI: 2.3–3.9) cases per 10 000 patient days and the monthly change in incidence during Period 1 was 0.05 cases per 10 000 patient days. After discontinuing contact precautions, there was no statistically significant increase in the incidence of healthcare-associated ESBL-producing *E. coli* (slope change Periods 2–4 combined: −0.04, *P* = .14). When considering each period (Figure 1*A*), we did not observe any statistically significant increases in the monthly change in incidence, or in the average incidence per period, during any of the Periods 2–4 as compared to Period 1 (Supplementary Tables 1 and 2). Similarly, when restricted to healthcare-associated ESBL-producing *E. coli* detected on clinical samples (Figure 1*B*), or in wards with systematic, weekly universal screening for antibiotic-resistant bacteria (Supplementary Figure 2), we also did not observe any statistically significant increases in the monthly change in incidence in any of the Periods 2–4 (Supplementary Table 1).

## DISCUSSION

In this retrospective analysis of inpatients at Geneva University Hospitals, we found no statistically significant increase in the monthly change in incidence of healthcare-associated ESBL-producing *E. coli* after the discontinuation of contact precautions for patients colonized or infected with ESBL-producing *E. coli*. The results were similar whether we considered the incidence of healthcare-associated ESBL-producing *E. coli* colonization or clinical infections and were consistent when restricted to wards in which weekly screening of all hospitalized patients was conducted throughout the study period.

From an infection control perspective, these findings are reassuring and align with the findings of a large cluster-randomized crossover trial [3] and with a systematic scoping review on the benefit of contact precautions in preventing healthcare-associated acquisition of ESBL-producing organisms [11]. Discontinuing contact precautions effectively reduced protective measures such as single-room isolation, the use of gloves and gowns, and enhanced cleaning and disinfection of the patient's environment. Despite this, we did not observe an increase in incidence of healthcare-associated cases. This is important, as contact precautions increase workload for healthcare workers and healthcare costs due to isolation and disinfection measures [5].

Although not directly assessed in this analysis, our findings support the assertion that community reservoirs may be a more important driver for the emergence and spread of ESBL-producing *E. coli* as compared to transmission within healthcare facilities [12, 13]. There has been a persistent increase in the proportion of carriers of ESBL-producing Enterobacterales, predominantly *E. coli* albeit with considerable geographic heterogeneity [14]. In the community, carriage of ESBL-producing *E. coli* is often linked to international travel to endemic countries, intrafamilial transmission or food consumption [6].

A strength of this analysis was the extended study duration, which captured both prior to and after the discontinuation of contact precautions, as well as two distinct periods during the COVID-19 pandemic. This likely reduced confounding from pandemic-related changes such as patient mix (fewer elective surgical procedures early in the pandemic), or infection control measures. Further, the analysis was conducted in a large tertiary care hospital with consistent screening strategies, microbiological testing and epidemiological surveillance throughout the study, reducing detection or reporting biases. In sensitivity analyses restricted to healthcare-associated ESBL-producing *E. coli* clinical infections, results were similar, further suggesting the absence of major detection bias.

Nonetheless, several limitations must be acknowledged. The discontinuation of contact precautions for patients colonized or infected with ESBL-producing *E. coli* was implemented hospital-wide on 1 January 2019. As such, we have a nonrandomized study design and no contemporaneous control group or counterfactual scenario. In addition, the analysis relied on aggregated surveillance data without individual-level information. Finally, the study was conducted in the context of a healthcare setting with consistently high compliance to standard precautions and hand hygiene (average annual observed healthcare worker compliance between 70% and 80%; data not shown). These findings may therefore not necessarily be generalizable to settings with lower infection prevention compliance.

Overall, in a large Swiss tertiary care hospital, the discontinuation of contact precautions for patients colonized or infected with ESBL-producing *E. coli* was not associated with an increase in healthcare-associated transmission.

## Supplementary Material

ciaf594_Supplementary_Data

## Supplementary Data

Supplementary materials are available at *Clinical Infectious Diseases* online. Consisting of data provided by the authors to benefit the reader, the posted materials are not copyedited and are the sole responsibility of the authors, so questions or comments should be addressed to the corresponding author.
