# Supplementary material for: Impact of Discontinuation of Contact Precautions on Healthcare-associated Extended Spectrum β-Lactamase-Producing Escherichia coli in a Large Tertiary Care Hospital: An Interrupted Time-series Analysis
Source: Clin Infect Dis. 2025 Oct 29;82(4):e794–8. doi: 10.1093/cid/ciaf594 (PMC13131913; doi:10.1093/cid/ciaf594)
Supplement: ciaf594_Supplementary_Data [file ciaf594_supplementary_data.docx]

**Impact of discontinuation of contact precautions on healthcare-associated ESBL-producing *Escherichia coli* in a large tertiary care hospital: an interrupted time-series analysis**

**Supplementary Material**


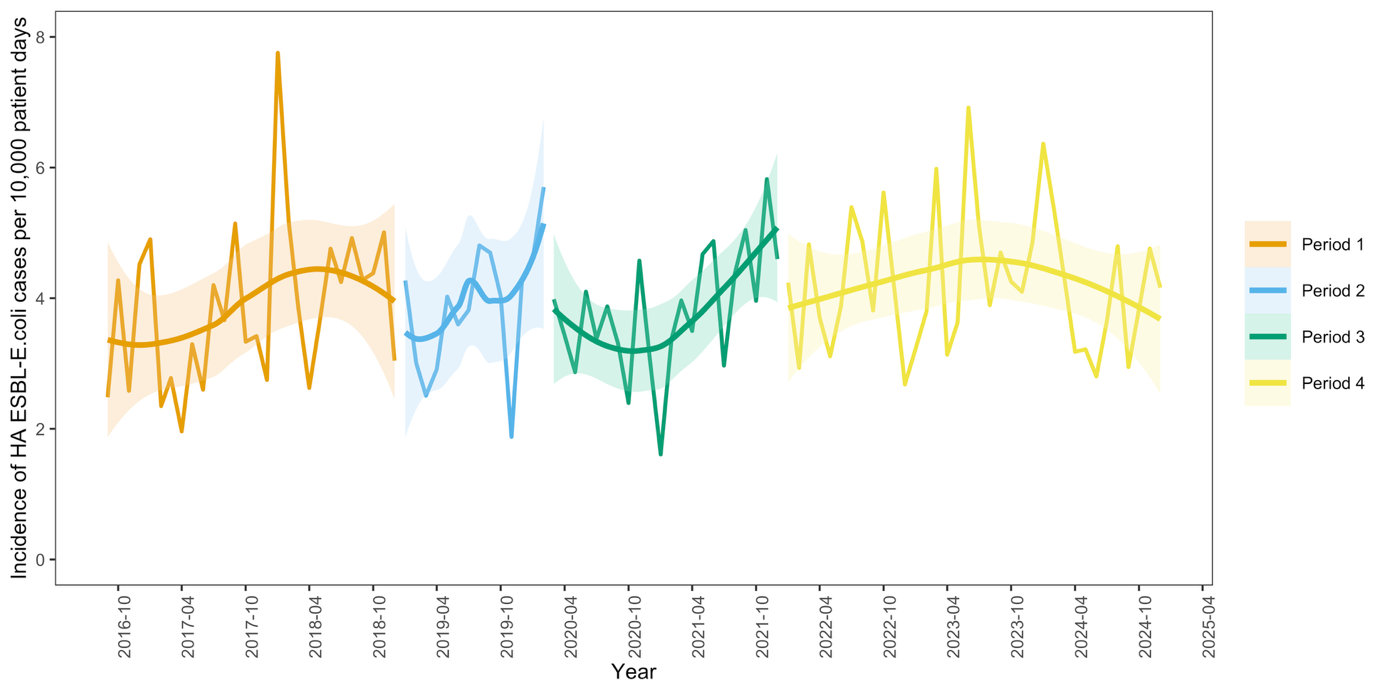


Supplementary Figure 1. Incidence of healthcare-associated ESBL-producing *E. coli*by period per 10,000 patient days (including both screening and clinical samples). The figure shows the LOESS smoothed trend, and the shaded area indicates the 95%CI.

Supplementary Table 1. Estimated monthly change in incidence of healthcare-associated ESBL-producing *E. coli*by period per 10,000 patient days

|  | Monthly incidence (per 10,000 patient days) | 95%CI | P value |
| --- | --- | --- | --- |
| A. All samples |  |  |  |
| Period 1: start | 3.1 | 2.3-3.9 |  |
| Period 1: slope | 0.05 | 0.0-0.1 | 0.04 |
| Relative to Period 1: |  |  |  |
| Period 2: slope | 0.06 | -0.09-0.2 | 0.42 |
| Period 3: slope | 0.02 | -0.06-0.1 | 0.63 |
| Period 4: slope | -0.05 | -0.10-0.01 | 0.10 |
| B. Clinical samples* only |  |  |  |
| Period 1: start | 1.5 | 1.1-1.9 |  |
| Period 1: slope | 0.02 | 0.0-0.1 | 0.04 |
| Relative to Period 1: |  |  |  |
| Period 2: slope | 0.02 | -0.04-0.1 | 0.51 |
| Period 3: slope | -0.03 | -0.08-0.1 | 0.12 |
| Period 4: slope | -0.01 | -0.04-0.02 | 0.37 |

*Clinical samples were considered as the detection of ESBL-producing *E. coli* more than 48 hours from the patient’s admission on any specimen type except rectal swabs, axillary swabs or fecal samples.

Supplementary Table 2. Incidence of healthcare-associated ESBL-producing *E. coli*by period per 10,000 patient days

| Period | Number of months in period | Number of healthcare-associated ESBL-producing *E. coli* | Number of patient days in period | Incidence per 10,000 patient days per period | 95%CI |
| --- | --- | --- | --- | --- | --- |
| Period 1 | 28 | 667 | 1,725,473 | 3.87 | 3.58-4.16 |
| Period 2 | 14 | 337 | 873,361 | 3.86 | 3.46-4.27 |
| Period 3 | 22 | 448 | 1,170,735 | 3.83 | 3.48-4.19 |
| Period 4 | 36 | 884 | 2,087,025 | 4.24 | 3.96-4.52 |


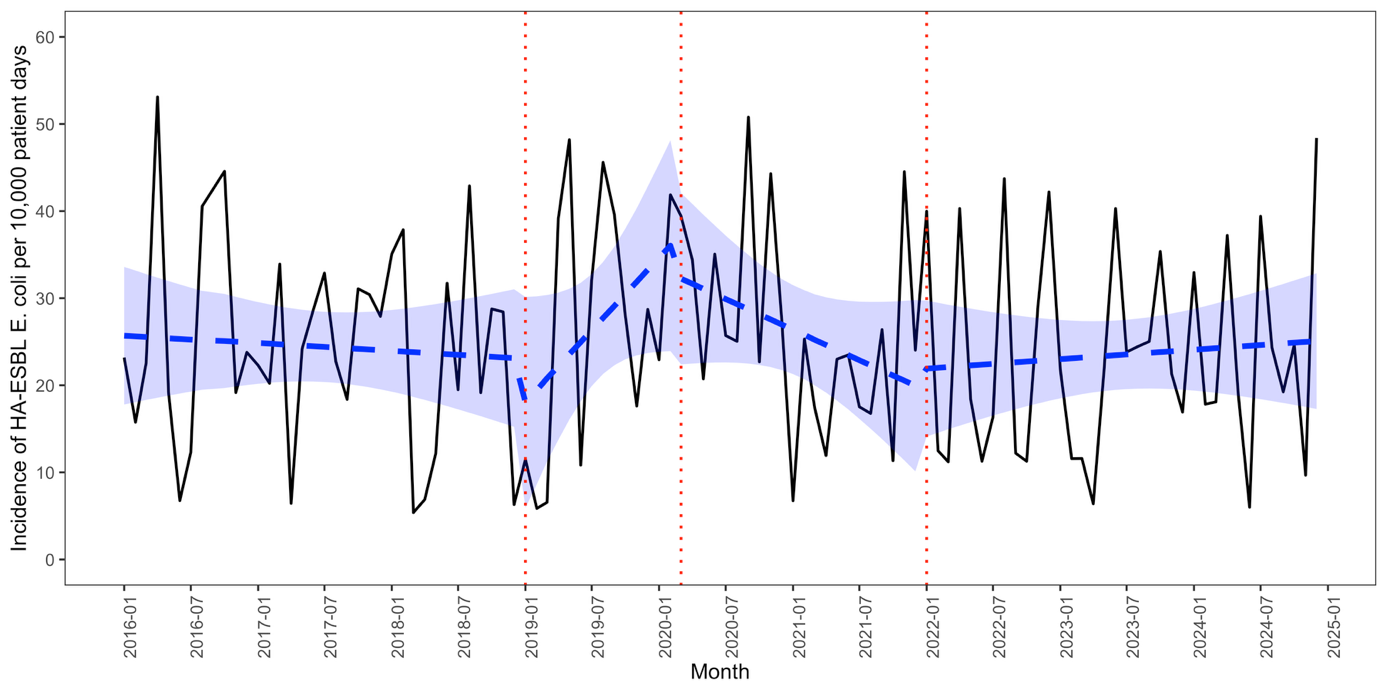


Supplementary Figure 2. Observed and fitted incidence of healthcare-associated ESBL-producing *E. coli* by period per 10,000 patient days on all samples in wards in which systematic, weekly screening of all hospitalized patients was conducted throughout the study period: adult intensive care, hemato-oncology and septic orthopedics. The red vertical lines distinguish each period: Period 1 (baseline), Period 2 (discontinued contact precautions), Period 3 (early COVID-19 pandemic) and Period 4 (later COVID-19 pandemic). The shaded area indicates the 95%CI of the fitted incidence.
